# Feasibility Study of the Permeability and Uptake of Mesoporous Silica Nanoparticles across the Blood-Brain Barrier

**DOI:** 10.1371/journal.pone.0160705

**Published:** 2016-08-22

**Authors:** Habib Baghirov, Didem Karaman, Tapani Viitala, Alain Duchanoy, Yan-Ru Lou, Veronika Mamaeva, Evgeny Pryazhnikov, Leonard Khiroug, Catharina de Lange Davies, Cecilia Sahlgren, Jessica M. Rosenholm

**Affiliations:** 1 Turku Centre for Biotechnology, University of Turku and Åbo Akademi University, Turku, Finland; 2 Laboratory of Physical Chemistry, Faculty of Science and Engineering, Åbo Akademi University, Turku, Finland; 3 Cell Biology, Faculty of Science and Engineering, Åbo Akademi University, Turku, Finland; 4 Department of Physics, Norwegian University of Science and Technology, Trondheim, Norway; 5 Pharmaceutical Sciences Laboratory, Faculty of Science and Engineering, Åbo Akademi University, Turku, Finland; 6 Centre for Drug Research, Division of Pharmaceutical Biosciences, University of Helsinki, Helsinki, Finland; 7 Neurotar LtD, Helsinki, Finland; 8 Eindhoven University of Technology, Eindhoven, The Netherlands; Universidad de Castilla-La Mancha, SPAIN

## Abstract

Drug delivery into the brain is impeded by the blood-brain-barrier (BBB) that filters out the vast majority of drugs after systemic administration. In this work, we assessed the transport, uptake and cytotoxicity of promising drug nanocarriers, mesoporous silica nanoparticles (MSNs), in *in vitro* models of the BBB. RBE4 rat brain endothelial cells and Madin-Darby canine kidney epithelial cells, strain II, were used as BBB models. We studied spherical and rod-shaped MSNs with the following modifications: bare MSNs and MSNs coated with a poly(ethylene glycol)-poly(ethylene imine) (PEG-PEI) block copolymer. In transport studies, MSNs showed low permeability, whereas the results of the cellular uptake studies suggest robust uptake of PEG-PEI-coated MSNs. None of the MSNs showed significant toxic effects in the cell viability studies. While the shape effect was detectable but small, especially in the real-time surface plasmon resonance measurements, coating with PEG-PEI copolymers clearly facilitated the uptake of MSNs. Finally, we evaluated the *in vivo* detectability of one of the best candidates, i.e. the copolymer-coated rod-shaped MSNs, by two-photon *in vivo* imaging in the brain vasculature. The particles were clearly detectable after intravenous injection and caused no damage to the BBB. Thus, when properly designed, the uptake of MSNs could potentially be utilized for the delivery of drugs into the brain *via* transcellular transport.

## Introduction

The blood-brain barrier (BBB) is the most extensive of barriers that protect the brain’s internal milieu and maintain its homeostasis [1]. Structurally, the BBB is formed by brain capillary endothelial cells (BCEC). While sharing some features with other endothelial cells, BCEC have a number of marked differences such as the structure of their tight junctions, lack of fenestrations, diminished pinocytosis, high mitochondrial activity, high percentage of proteins in the cell membrane and the expression of various BBB markers. Key components of the BBB—the paracellular barrier formed by circumferential tight junctions between adjacent BCEC and the transcellular barrier consisting of cell membranes, efflux transporters and various enzymatic filters–act together to form a dynamic interface that incorporates physical, metabolic and enzymatic mechanisms to screen the brain from harmful agents and ensure that its tightly controlled extracellular fluid microenvironment remains resistant to the much more volatile environment of blood [2, 3]. Unfortunately, this barrier function also makes the BBB filter out the vast majority of drugs, making the treatment of various brain disorders highly dependent on drug delivery limitations. The problem is widely acknowledged, and it has been estimated that 100% of large molecules (over 500 Da) and 98% of small molecules do not reach the brain after systemic administration, making the central nervous system drug market largely underpenetrated [4].

Nanoparticles, due to their high drug load capacity and possible functionalization for facilitating BBB permeability, as well as imaging and targeting, have emerged as a possible solution to this challenge [5–7]. They come in a variety of sizes and shapes and can be further tailored to desired needs by surface modification. They can carry many drug molecules without requiring chemical modification of the same, which is important for preserving drug activity. Unlike traditional drug formulations, where drug release is spontaneous and immediate, often requiring frequent administrations, drug delivery using nanoparticles can be controlled and sustained, thus increasing target availability. Both qualities may further be enhanced by functionalization, e.g. by capping porous particles with ‘gatekeepers’ or using cleavable agents, respectively. In addition, nanoparticles can be bound to ligands or antibodies for active targeting, which can decrease non-specific toxicity of drugs by indirectly reducing their levels in non-targeted tissues.

One class of inorganic nanoparticles that has distinct advantages in drug delivery is mesoporous silica nanoparticles (MSNs) [8–10]. MSNs have a well-defined pore arrangement with controllable pore sizes in the 2–50 nm range. The pores can occupy a large part of the total volume, and their design can be adjusted to accommodate drugs, imaging agents or both. Aside from endowing MSNs with a large surface area, porosity allows independent functionalization of inner and outer surfaces. The former can improve drug immobilization, while the latter can be used for better stability in suspension, controlled or more sustained release (e.g. by capping pore openings), charge modification, and linkage to targeting ligands, hydrophilic moieties etc. The porous structure also helps to physically separate cargo in multifunctional nanoparticles. Generally, drugs are loaded into the pores for better protection and immobilization, while various moieties, as well as imaging and targeting agents are attached to the outer surface. At neutral pH, unmodified MSNs are negatively charged, whereby their functionalization with cationic surface groups can be used for enhanced accommodation of negatively charged (e.g. acidic) drugs, but also for promoting endosomal escape [11]. MSNs are thermally and chemically stable; eventually, however, their matrix undergoes biodegradation by hydrolysis. Degradation of MSNs produces beneficial monomeric silicic acid [12], and this increases the appeal of MSNs compared to other inorganic nanoparticles, many of which are not biodegradable.

Given these potential advantages of MSNs in drug delivery, we decided to investigate whether MSNs can cross or be taken up by the BBB and whether this uptake or permeability is affected by their 1) aspect ratio or 2) the effect of a poly(ethylene glycol)-poly(ethylene imine) (PEG-PEI) block copolymer layer, of which the PEG component is commonly used to reduce the recognition of circulating particles by the reticuloendothelial system *in vivo*, thus also increasing the likelihood of BBB penetration [6], and the PEI component confers cationic properties, thus facilitating cellular uptake and, potentially, permeability [13]. One of the more promising candidates was selected for an *in vivo* detectability test. In addition, we evaluated cytotoxic effects of the studied MSNs. Three complementary methods were used to evaluate cellular uptake: flow cytometry, confocal fluorescence microscopy and surface plasmon resonance (SPR). SPR is a novel, label-free method for studying nanoparticle interactions with cells where the interactions can be followed in real time, and our observations were thus correlated with those of the more traditional methods (flow cytometry and microscopy). Further, we demonstrated the detectability of the developed particles in mice by applying two-photon *in vivo* imaging. We used RBE4 rat brain endothelial cells and Madin-Darby canine kidney epithelial cells, strain II, as BBB models; RBE4 cells are of brain endothelial origin, thus reflecting the BBB more closely, but MDCK II, while of kidney epithelium origin, may be preferable in transport studies as they form a monolayer with considerably better barrier properties.

## Results and Discussion

### MSN synthesis and design

**Fig 1:**
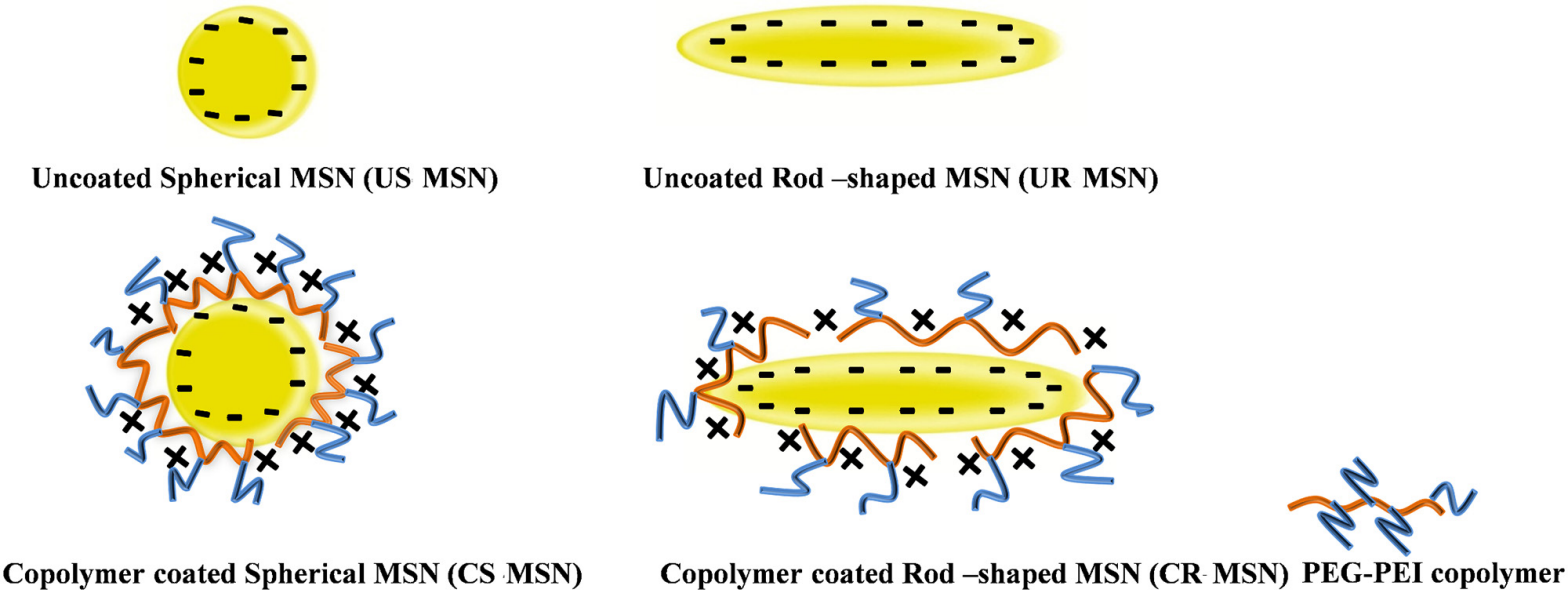
Particle designs implemented for this study.

In order to obtain particles in the sub-100 nm range, MSNs were synthesized according to the procedure described by Gu et al. [14] with certain modifications to further vary the aspect ratio of the resulting particles. The synthesis parameters were thus varied in order to investigate the effect of aspect ratio, where rod-shaped MSNs have previously been observed to be more efficiently internalized by cells [7, 15, 16]. In addition, rod-shaped particles have in certain cases proven to be more efficient in permeability studies [2, 3], which is why we set out to investigate whether we could detect any favorable effects related to aspect ratio in our case as well. The synthesized particles were further coated with in-house produced PEG-PEI copolymers [17] which could, if desired, be further attached to biomolecular moieties. All four particle designs are depicted in Fig 1.

**Fig 2:**
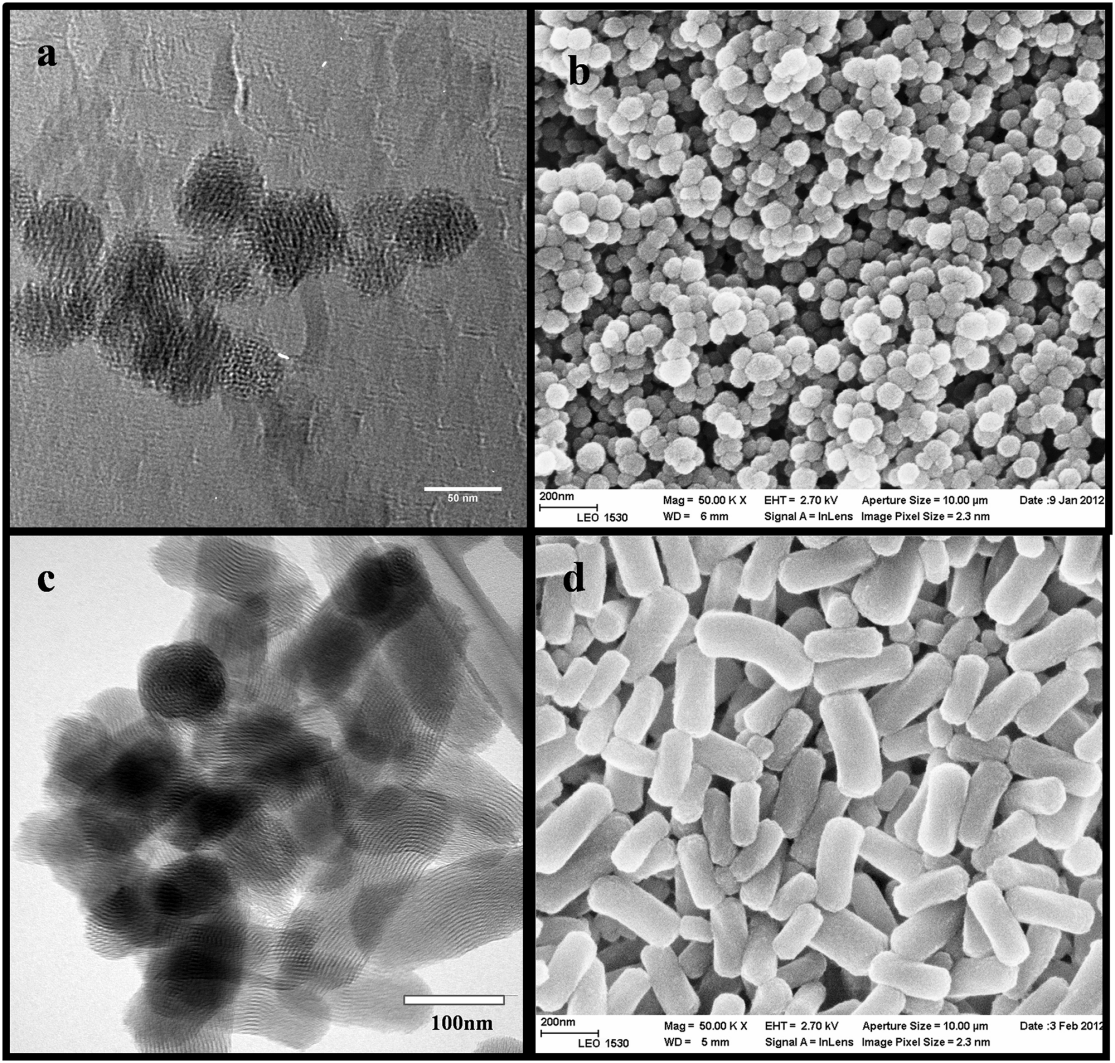
Electron microscopy images. a) transmission electron microscopy images of uncoated spherical MSNs b) scanning electron microscopy images of uncoated spherical MSNs c) transmission electron microscopy images of uncoated rod-shaped MSNs d) scanning electron microscopy images of uncoated rod-shaped MSNs.

After template extraction, the morphology and mesostructure of the obtained pure spherical MSN and rod-shaped MSNs were investigated with electron microscopy (EM), as shown in Fig 2 below.

The coated PEG-PEI copolymer amount on both spherical MSN and rod-shaped MSN was about 13 wt% for both particle types as determined by thermogravimetric analysis (TGA). For all particle types, suspensions were prepared in concentrations of 1 mg/ml in HEPES buffer (pH 7.2), and hydrodynamic size and net surface charge (ζ-potentials) were determined by using dynamic light scattering and electrokinetic analysis, respectively. Fluorescence intensity spectra were recorded and the peak value at the highest emission wavelengths upon excitation at 488 nm was noted. These maximum peak values as well as dynamic light scattering (DLS) and ζ-potential data can be found in Table 1 below.

**Table 1:** Physicochemical characteristics of the investigated particle suspensions in HEPES buffer (pH = 7.2).

Samples	Hydrodynamic size (nm)	Net surface charge (mV)	Fluorescence intensity
**Uncoated spherical MSN (US MSN)**	152	-24	501
**Coated spherical MSN (CS MSN)**	137	8	23
**Uncoated rod-shaped MSN (UR MSN)**	207	-27	439
**Coated rod-shaped MSN (CR MSN)**	222	10	79

Clearly, all particles were fully dispersible in HEPES buffer after all processing steps, including template removal and surface coating with copolymer. We note that the hydrodynamic size derived from DLS measurements is not used to establish a “real” particle size but rather dispersability, whereby EM (Fig 2) is applied for particle size determination. On the one hand, particle fluorescence may distort the DLS values obtained from such a measurement, and on the other hand, particles with non-spherical morphology cannot be size-determined using light scattering methods. Thus, according to the EM images, the uncoated spherical MSNs have spherical morphology with a diameter of 50 nm and uncoated rod-shaped MSNs have elongated morphology with the aspect ratio of 3 (length~ 300 nm width ~100 nm). The ζ-potentials at neutral pH confirmed that both particles in question were comparable to pure silica in terms of net surface charge (despite the addition of small amounts of aminosilane for the covalent attachment of the fluorescent label) and thus applicable for electrostatic coating of the produced PEG-PEI copolymer. In this specific copolymer construct, the cationic PEI part is used to anchor strongly to the negatively charged silica surface, whereas the PEG chains are expected to ‘stick out’ from the particle surface and thus impart the particle system with a steric stabilization component [17]. Further, PEG is probably the most commonly employed polymer coating for nanomedical systems especially with *in vivo* prospects. The electrostatic contribution of PEI could be clearly observed in the electrokinetic measurements, with a shift from the characteristic negatively charged surface of silica to positive at neutral pH (Table 1). The proximal location of PEI was also observed as a decrease in fluorescence intensity, where the local abundance of protons, owing to the ‘proton trap’ ability of PEI, decreases the local pH which, in turn, decreases the quantum yield and thereby the resulting fluorescence intensity of fluorescein [18].

### Cytotoxicity studies

Cytotoxicity experiments were performed with both MDCK II and RBE4 cells and were based on the viability of cells following incubation with spherical and rod-shaped of MSNs for 36 hours.

**Fig 3:**
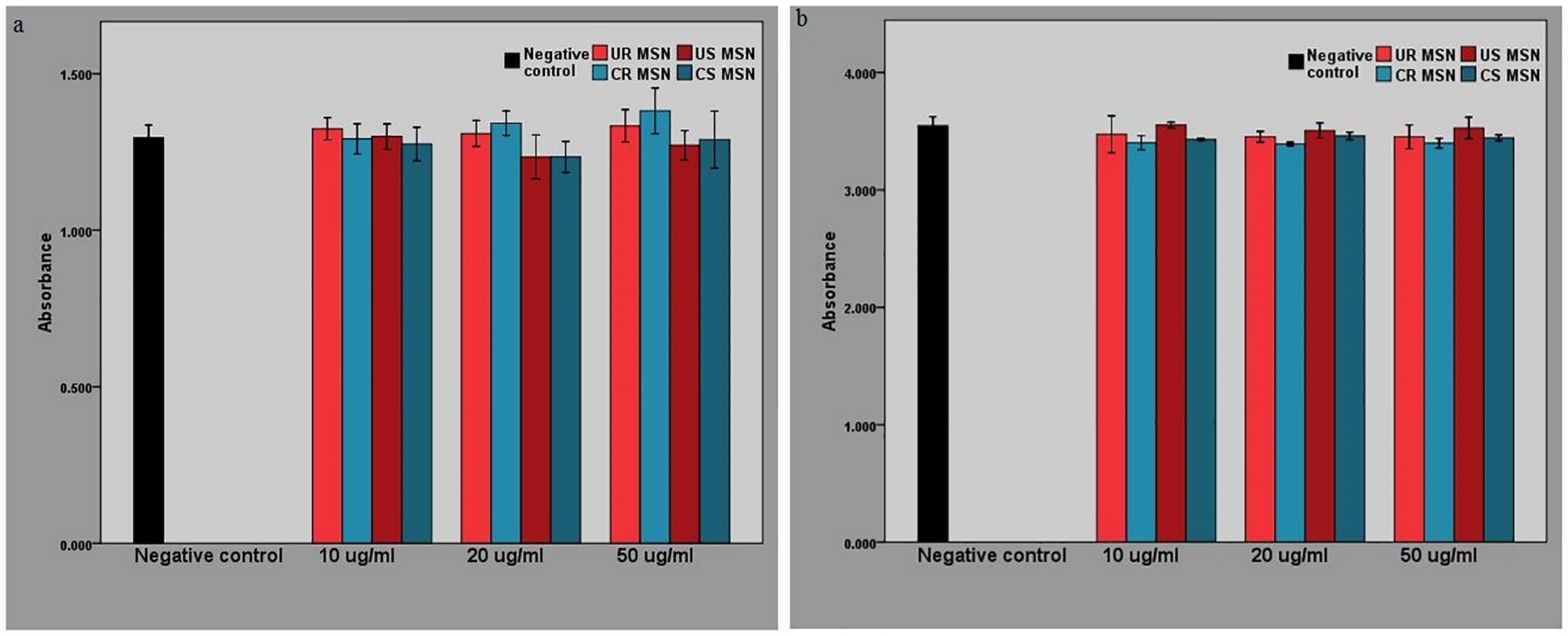
Cytotoxicity of various MSNs applied at concentrations of 50, 20 and 10 μg/ml in serum-free medium. Negative control–untreated cells. Data shown as M±2xSEM. **a)** MSN toxicity in MDCK II cells. **b)** MSN toxicity in RBE4 cells.

As can be seen in Fig 3a and 3b, neither rod-shaped, nor spherical MSNs show any considerable toxic effect at concentrations of 50 μg/ml and below. The relatively long incubation time in the cytotoxicity experiments has allowed us to verify that MSN NPs at the studied concentrations were did not have any toxic effect in either cell line at any of the time points used in other experiments in our study.

Additionally, bright field microscopy images of MDCK II monolayers used in transport experiments were taken in order to estimate the toxic effect of the MSNs on fully polarized MDCK II cells.

**Fig 4:**
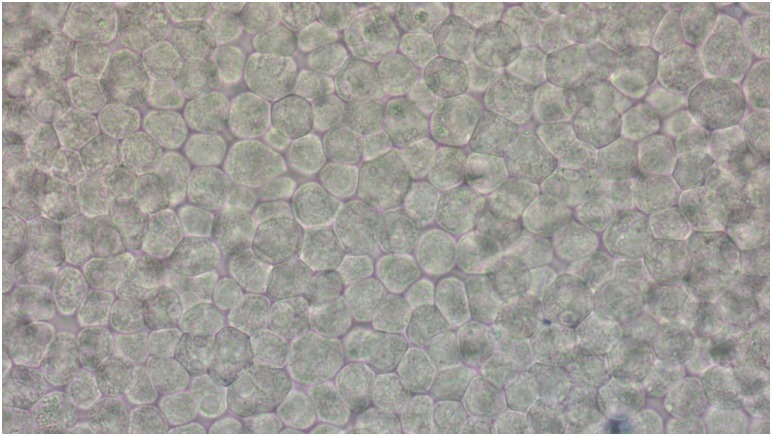
Bright field microscopy image of MDCK II cells grown on permeable supports and incubated with rod-shaped MSNs coated with a PEG-PEI copolymer (incubation time—36 hours).

In Fig 4, few to no cells stained by Trypan Blue are seen on a permeable support with PEG-PEI-coated rod-shaped MSNs (chosen here as an example), which indicates lack of a substantial toxic effect. No signs of monolayer disruption were observed in the bright field images of monolayers exposed to other MSNs (data not shown).

Having established that our MSN do not exert any toxic effect at concentrations up to 50 μg/ml, we proceeded with uptake and transport studies using the nanoparticles in the same range of concentrations.

### Nanoparticle uptake in cellular models *in vitro*

As nanoparticles in general are too large to pass through the tight cell junctions of an intact BBB (paracellular route), the expected mechanism of transport over a cell layer would rather be transcytosis (transcellular route). In order for this to take place, the particles would first have to be taken up by the cells via endocytosis, be translocated across the cell, and, ultimately, exocytosed. Thus, MSN uptake was evaluated by flow cytometry and confocal microscopy to detect fluorescein isothiocyanate (FITC)-labeled MSNs taken up by cells and, in a novel approach, by surface plasmon resonance measurements which allow monitoring cell uptake of nanoparticles in real time without using labels.

**Fig 5:**
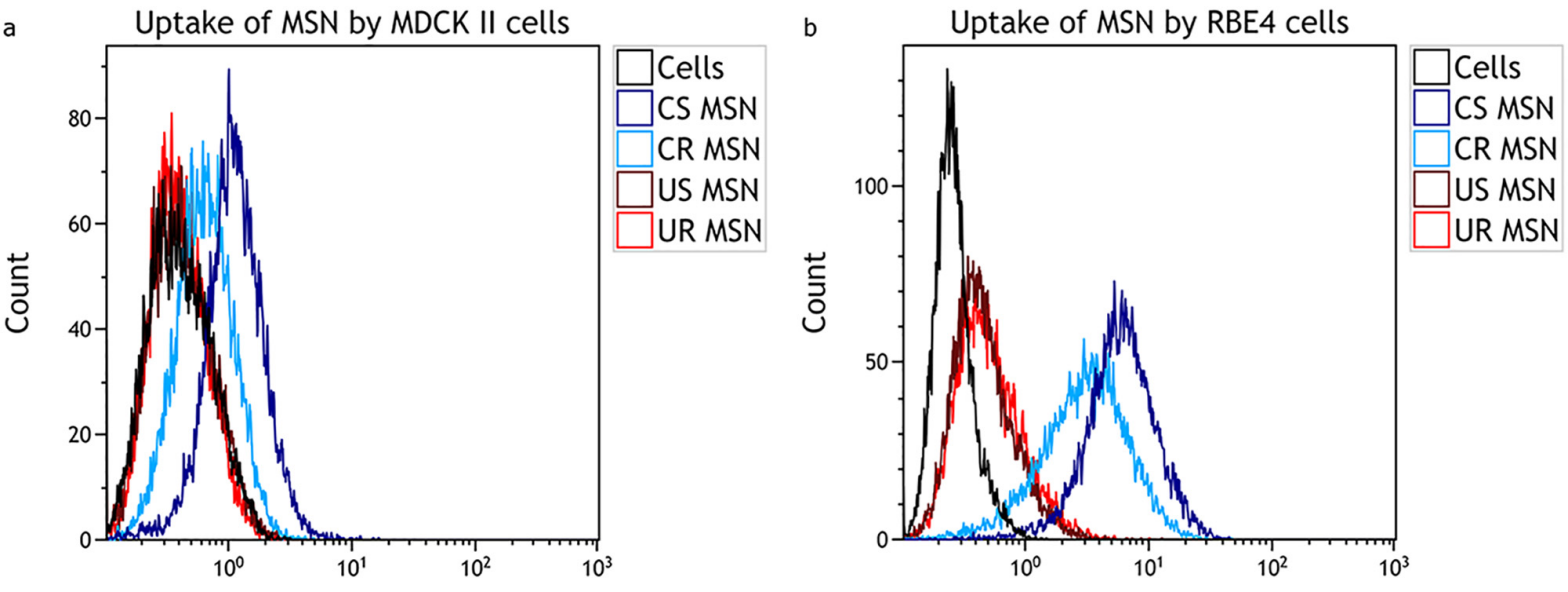
Flow cytometry histograms for determining the uptake of different MSNs that were incubated with the MDCK II and RBE4 cells at a concentration of 20 μg/ml for 24 hours in serum-free medium. Negative control–cells not incubated with the MSNs. a) Representative histograms showing uptake of MSNs in MDCK II cells. b) Representative histograms showing uptake in RBE4 cells.

As shown in Fig 5a and 5b, the uptake of copolymer-coated spherical and rod-shaped MSNs in both MDCK II and RBE4 cells is robust and manifested by complete peak shifts in their respective histograms, indicating that the copolymer coating was very efficient in improving the cellular uptake of both spherical and rod-shaped MSNs. The uptake of uncoated particles is much less prominent, even though some uptake can be detected as judged by a fraction of FITC-positive cells, seen as a ‘bulging’ in the histogram.

Problems related to the fluorescence variability of the label used (fluorescein) lead us to refrain from drawing any conclusions regarding quantification based on flow cytometry studies. In order to further confirm MSN uptake results, however, we evaluated the internalization of MSNs using confocal microscopy.

**Fig 6:**
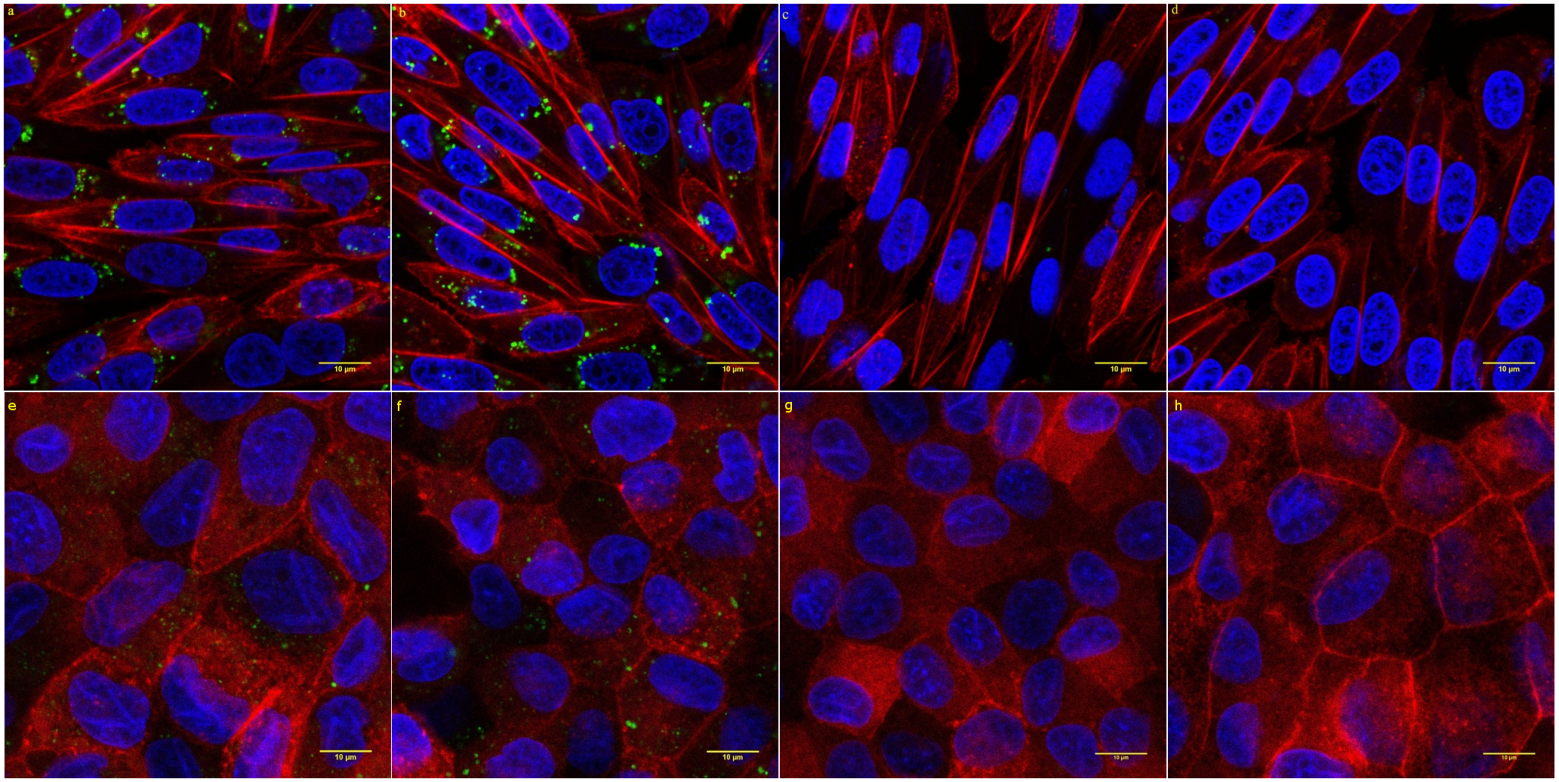
Confocal images of RBE4 and MDCK II cells incubated for 24 hours with MSNs applied at a concentration of 20 μg/ml in serum-free medium. Nuclei stained with Hoechst 33258 (blue). MSNs labeled with FITC (green). F-actin stained with phalloidin (red) **a)** PEG-PEI-coated spherical MSNs in RBE4. **b)** PEG-PEI-coated rod-shaped MSNs in RBE4. **c)** Uncoated spherical MSNs in RBE4. **d)** Uncoated rod-shaped MSNs in RBE4. e) PEG-PEI-coated spherical MSNs in MDCK II. f) PEG-PEI-coated rod-shaped MSNs in MDCK II. g) Uncoated spherical MSNs in MDCK II. h) Uncoated rod-shaped MSNs in MDCK II.

Fig 6a–6d show the uptake of MSNs by RBE4 cells. Only few uncoated MSNs are visible inside RBE4 cells, while the uptake of PEG-PEI-coated MSNs is very robust, which is in line with the flow cytometry data. Many PEG-PEI-coated MSNs are found around the cell nuclei, which can be explained by the long incubation time used in this experiment as the nanoparticles located in endocytic pathway vesicles would have enough time to shift toward the nuclei along microtubules in a retrograde transport. Images of MDCK II cells incubated with MSN NPs show the same trends, although the uptake efficiency is lower, which is consistent with the flow cytometry results. Overall, confocal microscopy results support the importance of PEG-PEI coating for cellular uptake of MSNs.

In addition to the more established techniques such as FCM and CLSM, we also employed SPR as a novel method to study the MSN-cell interactions in greater detail in order to determine possible differences between uncoated and polymer-coated MSNs. The advantages of SPR include monitor uptake in real time, independence of fluorescent labeling and great sensitivity. In the SPR, the signal response is caused by a combination of morphological changes in the cell layer, i.e. cell spreading or contraction, and accumulation of the stimulant in the cells [19–21] The SPR response should thus reflect the endocytotic uptake of nanoparticles and depend on the translocation of MSN-containing vesicles from the membrane.

**Fig 7:**
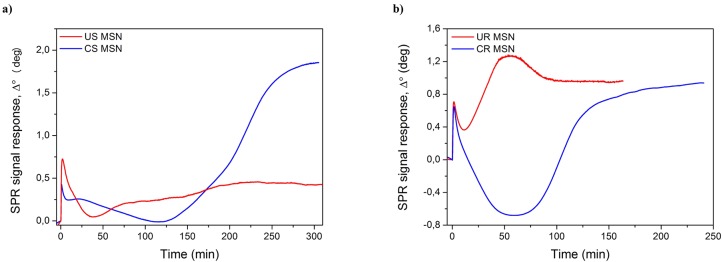
SPR responses reflecting uptake of MSNs by MDCK II cells. Cells were stimulated with 20 μg/ml of a) spherical and b) rod-shaped MSNs in serum-free medium at t = 20°C. Monitoring was performed for 175–240 minutes for spherical MSNs and 200–300 minutes for rod-shaped MSNs after injection of MSNs into a steady state SPR cuvette.

Fig 7 represents the interactions of spherical and rod-shaped MSNs with MDCKII cells at a concentration of 20 μg/ml. The initial peak during the first minutes mainly originates from the perturbations caused by the particle stock suspension media (DMSO) and can therefore be disregarded. In Fig 7a, for the copolymer coated spherical MSNs (blue curve) the initial leveling out of the SPR response (7–18 minutes) is interpreted to be caused by the fast adherence of the positively charged MSNs to the cell surface, causing a slight cell spreading and cytoskeletal mass re-distribution within the cell closer to the basolateral side of the cells [19, 20]. The subsequent decrease in the SPR signal (18–125 minutes) is then caused by cell contraction accompanied with cytoskeletal mass re-distribution towards the apical side of the cells during endocytosis of the copolymer coated spherical MSNs [19, 21]. This is then followed by a steep increase and leveling out of the SPR response (125–300 minutes) indicating continued cell uptake and subsequent intracellular translocation of the MSNs closer to the sensor surface, i.e. closer to the basolateral side of the cells. For the uncoated MSNs (red curve) the initial morphological change accompanied with cytoskeletal mass re-distribution towards the apical side of the cells during endocytosis seen as a decrease in the SPR signal takes place between 7–35 minutes. This is then followed by a much slower increase and leveling out of the SPR signal (35–300 minutes) and a much slower uptake and intracellular translocation kinetics compared to the copolymer coated spherical MSNs.

Different behavior can be observed for the rod-shaped MSNs in Fig 7b. The uncoated rod-shaped MSNs (red curve) induce an immediate steep increase in the SPR response (10–45 minutes) followed by a leveling out (45–55 minutes) and subsequent decrease and a second leveling out of the SPR response (55–175 minutes). This is interpreted as an immediate and very rapid uptake of the uncoated rod-shaped MSNs by the MDCK II cells accompanied with cytoskeletal mass re-distribution towards the apical side of the cells during endocytosis. With time, the uptake and intracellular translocation processes of the uncoated rod-shaped MSNs then reach a saturation point. However, the copolymer-coated rod-shaped MSNs initially display a deep decline onto the negative side in the SPR response (blue curve) followed by a steep increase and finally a leveling out in the SPR response. We again assign the initial substantial decrease in the SPR response as morphological changes in the cells accompanied with cytoskeletal mass re-distribution towards the apical side of the cells during endocytosis. These morphological and mass re-distribution changes are clearly more pronounced for the copolymer-coated rod-shaped MSNs compared to the spherical MSNs which is logical due to the different shape and size of the rod-shaped MSNs compared to the spherical MSNs. The steep increase in the SPR response towards later time points further indicates a continued and efficient uptake and intracellular translocation of the coated rod-shaped MSNs closer to the basolateral side of the cells, similarly as seen for the coated spherical MSNs.

### Nanoparticle transport across cellular monolayers *in vitro*

Having established robust uptake of PEG-PEI-conjugated nanoparticles, we evaluated the transport rates of rod-shaped and spherical MSNs across MDCK II monolayers. MDCK II monolayers were incubated with 50 μg/ml MSNs in serum-free medium, with their subsequent detection in the samples of the basolateral compartment taken at different time points. MDCK II cells were chosen as a BBB model in the transport study because of their barrier properties that are considerably higher than those of RBE4 cells. Detection was based on fluorescence emitted by the fluorescent tag (FITC) used to label the MSNs.

**Fig 8:**
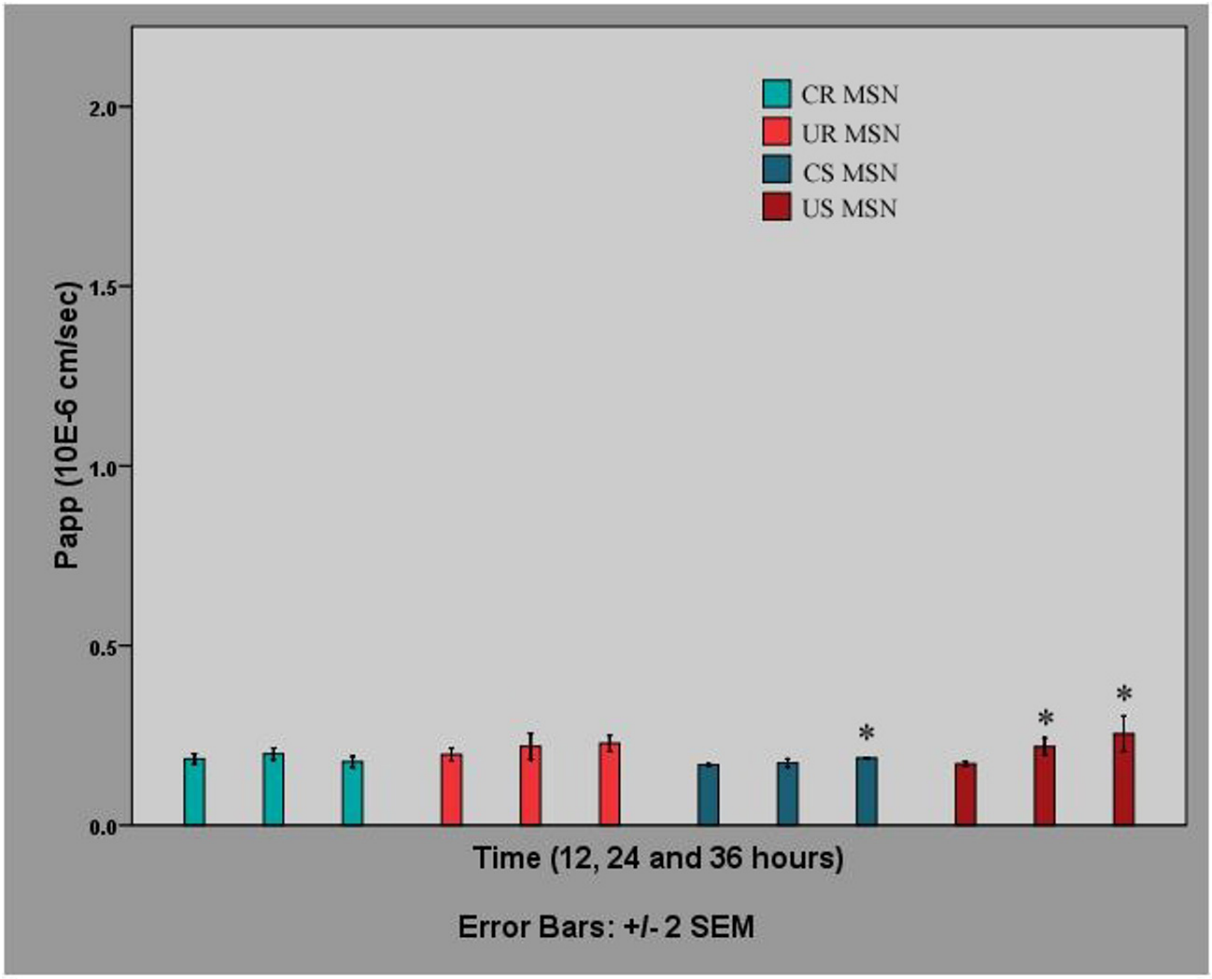
Transport of different MSNs across MDCK II monolayers in serum-free medium. MSNs were applied at a concentration of 50 μg/ml. Data represent the mean apparent permeability (Papp) of MSN (n = 3) at the time points 12, 24 and 36 hours, corrected for the loss of MSNs in the upper compartment, and are shown as M±2xSEM.Asterisks denote significant differences between Papp at 24 or 36 hours and Papp at 12 hours (p<0.05).

As shown in Fig 8, the transport of different MSNs across an MDCK II monolayer is generally low at the studied concentration, with the slight exception of the transport of spherical MSNs at 36 hours. Changes in the transport of rod-shaped nanoparticles over time are not statistically significant; the increase in the transport of spherical nanoparticles, especially in the case of uncoated MSNs, is significant, but very small. We note that particle concentrations used in similar transport studies have generally been significantly higher, with concentrations of 176 μg/ml [22] and 224 μg/ml [23] reported for polymeric particle, and in the latter the same concentration for metal oxide nanoparticles, whereas concentrations of 1.0–1.5 mg per monolayer have been used specifically for silicon-based particles [24], which could partly explain these results. However, in order to avoid toxic effects, which may certainly also lead to increased permeability observations in this setup, we opted to stay within reasonable particle concentrations (up to 50 μg/ml in the present case). Cell viability evaluation was also carried out to ensure the safety of the used concentrations (see above).

### Nanoparticle detectability and BBB integrity *in vivo*

Having established that the MSNs were safe, *in vivo* two-photon microscopy was applied to investigate the *in vivo* detectability of rod-shaped PEG-PEI-conjugated MSNs, which we considered one of the most promising candidates on the basis of the previous uptake experiments. MSNs were injected i.v. into the tail vein of a mouse (*n* = 1).

**Fig 9:**
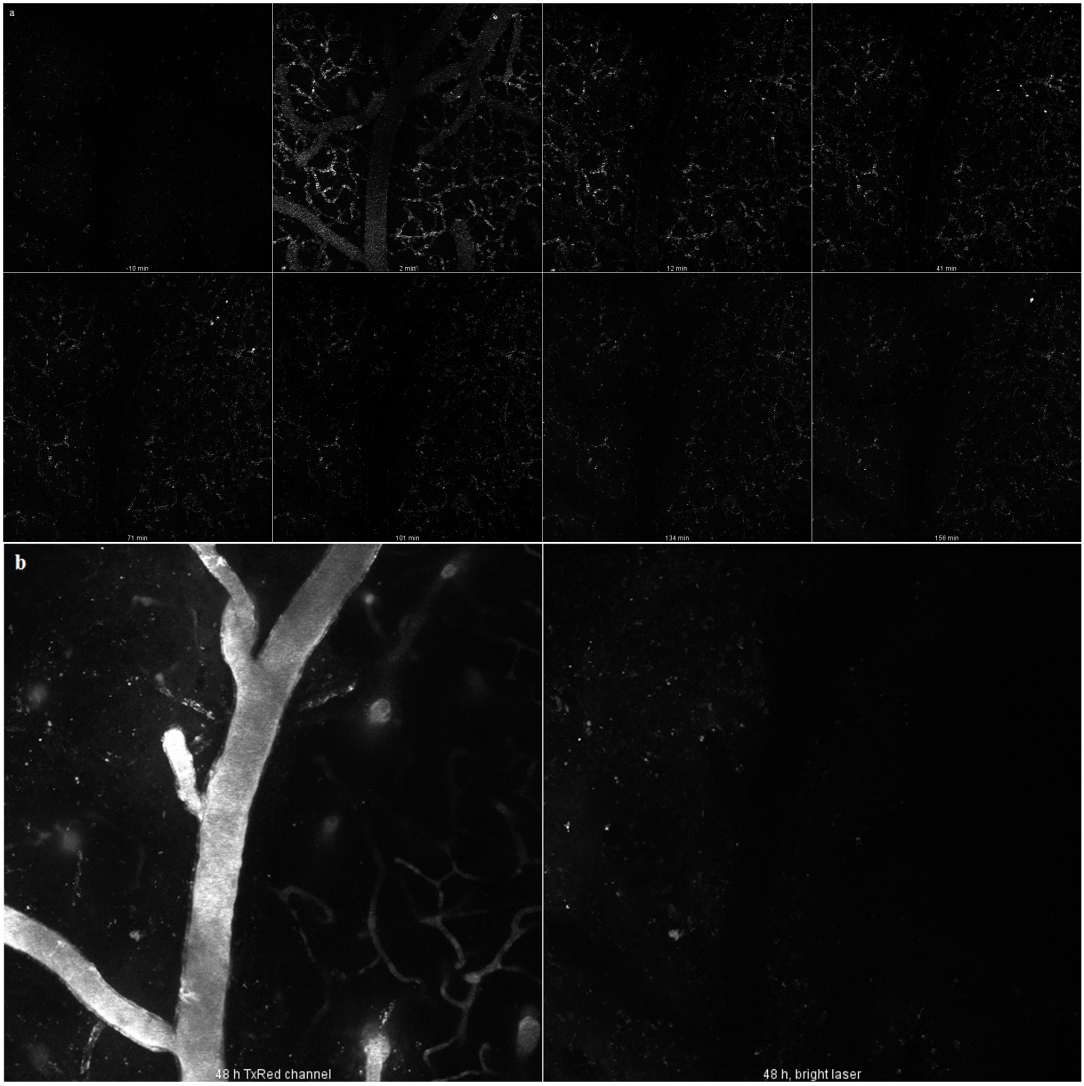
Brain distribution of rod-shaped MSNs after injection into the tail vein of a mouse. **a)** Rod-shaped MSNs imaged at different time points up to 156 minutes. **b)** Images taken at the end of the experiment (48 hours), showing brain vessels visualized with FITC-dextran, as well as the remaining red-shaped MSNs.

The particles were clearly detectable in circulation within minutes of i.v. injection. Very quickly, the number of particles in the vessel lumen decreased, and for the rest of the first imaging session they remained only on the vessel walls. No penetration through BBB was evident in this experiment, which is consistent with our *in vitro* results. At 48 hours, very few if any particles were observed, despite increasing the laser intensity by a factor of three. At the end of this second imaging session, a fluorescent dextran was injected to visualize the vessels and test BBB integrity: both appeared intact (see Fig 9).

Despite showing no BBB penetration for this particular nanoparticle species, the results clearly show that the particles can be visualized in vessels and be quantified if desired (by counting the actual number of particles in each compartment at each time point). This will be a valuable tool for developing particles with optimized desired properties.

The dimensions of the MSNs used in our study (ranging from 50 to 240 nm) would make their paracellular transport across a cell monolayer with fully formed tight junctions unlikely, therefore restricting their passage to the transcellular route or, more specifically, to endocytosis and subsequent exocytosis. So far, research into transcytosis of nanoparticles has produced varying results. On the one hand, it has been observed in numerous studies [25–28]. In [29], the authors found that the exocytosis of transferrin-coated gold nanoparticles in HeLa cells was even faster than endocytosis, especially for small nanoparticles. In many other studies, however, transcytosis of internalized nanoparticles was shown to be low to inexistent [24, 30]. One important factor could be incubation duration: e.g. in [31], the authors determined two stages of translocation: slow lag state phase (up to 8 hours) and steady state phase (9–27 hours). It should also be noted that many studies rely on fluorescent tags for the detection of exocytosed nanoparticles; this may interfere with the interpretation of results due to particle dissolution (with subsequent release of the dye) and pH-dependent fluorescence of certain dyes (e.g. FITC in our study). This limitation is seldom acknowledged [32], and studies involving natively fluorescent (quantum dots, nanodiamonds), luminescent (gold), paramagnetic (ferrous oxide) and otherwise detectable nanoparticles offer, in this sense, a distinct advantage.

We are aware of several studies where silica (not necessarily mesoporous) nanoparticles were reported to cross the BBB. In one study, FITC-labeled and TAT-conjugated silica particles were detected in the brain after intra-arterial administration [33]. In another, FITC-labeled silica nanoparticles were reported to cross static hCMEC/D3-based BBB model; however, while the uptake of nanoparticles was confirmed, transport rates were very low, and the authors acknowledged potential issues with dye leakage [32]. In a subsequent study, the same group found extensive evidence of silica nanoparticle endocytosis, but very few instances of their transcytosis in an hCMEC/D3-based BBB model. A similar study [34] found that 30 nm, but not larger silica nanoparticles crossed a commercial *in vitro* BBB model based of triple co-culture of primary rat endothelial cells, pericytes and astrocytes. The detection was based on a fluorescent marker; the authors did not acknowledge potential nanoparticle degradation issues. In [35], silica nanoparticles were not found in the brain of rats within 90 days after oral and dermal administration; a similar study conducted on mice, did not find silica nanoparticles the brain within 10 hours after oral exposure [36]. In [37], magnetic nanoparticles overcoated with silica were detected in the brain after intraperitoneal administration. That study spanned four weeks and the first time point was week 1; uptake by neurons was confirmed immunofluorometrically. In [38], organically modified “ORMOSIL” silica nanoparticles were found to be internalized by neurons in Drosophila; however, in an earlier biodistribution study on mice, the same nanoparticles did not accumulate in the brain [39]. In [40], PEGylated silica nanoparticles were found to cross the BBB both *in vitro* (in a model based on bEnd3 cells) and *in vivo* in athymic BALB/c mice; nanoparticle detection both *in vitro* and *in vivo* was based on a fluorescent signal from a doped Rubpy dye. In [41], PEGylated polyamidoamine dendrimer-conjugated magnetic MSNs were internalized by rat BCEC, but also by astrocytic endfeet and neurons (indicating transcytosis through BCEC); the authors observed no permeability of pure MSNs. To the best of our knowledge, it is the only study in which MSN-based nanoparticles were shown to cross the BBB in mammals. We also note that the design of this particle was very similar to ours, with sub-100 nm particles with a mesoporous silica coating to which PEG chains were anchored via a mediating polyamine layer, further emphasizing that the copolymer layer may be feasible for BBB crossing.

Assuming that the uptake study results do indicate more efficient uptake of coated MSNs compared to pure MSNs, this would be consistent with conclusions made by other authors in studies on cationic MSNs or many other types of nanoparticles. The size of the MSNs we used is also favorable to their internalization, and while coating may slightly increase the size of an individual particle, it reduces particle aggregation. We can assume that the PEG-PEI copolymer used for coating MSNs improved nanoparticle internalization mostly due the PEI component conferring positive charge, as PEG has not been shown to significantly improve nanoparticle uptake in vitro, unlike *in vivo* scenarios where it is used for screening nanoparticles from the reticuloendothelial systems, thus increasing their availability.

It should be noted that surface plasmon resonance measurements have not, to our best knowledge, yet been used for real-time monitoring nanoparticle uptake in cells. Thus, it is a novel method of monitoring endocytosis of nanoparticles and, therefore, as no comparative literature exists, the interpretation of the SPR results is best supported by alternative techniques such as flow cytometry or permeability studies. The large difference between the SPR responses for the spherical MSNs at 300 min suggests a higher uptake of the copolymer-coated particles compared to the uncoated particles, which is consistent with the flow cytometry results. The difference in the cell uptake kinetics of the uncoated and copolymer-coated spherical MSNs seen in the SPR responses could indicate that the different nanoparticles are taken up by the cells through different endocytotic mechanisms or have different intracellular trafficking routes. For example, the lower and slower increase in the SPR signal in the case of uncoated MSNs could indicate that more of these nanoparticles end up in transcytotic vesicles and recycling endosomes compared to copolymer coated spherical MSNs, whereas the copolymer-coated spherical MSNs with their ‘proton trap’ ability originating from PEI mainly end up in the lysosomes or multivesicular bodies and remain in the cells. This scenario is supported by the permeability studies which shows that the uncoated spherical MSNs has a higher *P*_app_ value compared to the polymer coated spherical MSNs. Similar interpretation can be suggested for the rod -shaped MSNs. Even though the *P*_app_ values are in the same order of magnitude and the absolute SPR response levels out at similar values for both rod-shaped MSNs, the cell uptake kinetics, reflected in the shape of the SPR curve, is clearly different between the uncoated and copolymer coated rod-shaped MSNs. According to the flow cytometry studies there is a clearly larger cell uptake of copolymer coated rod-shaped MSNs compared to uncoated rod-shaped MSNs. Thus, the SPR responses for the rod-shaped MSNs should be interpreted so that the cell uptake of the nanoparticles is reflected in the difference between the minimum and maximum responses during cell stimulation with the rod-shaped MSNs. Using this kind of interpretation of the SPR kinetics curve clearly shows that the copolymer coated rod-shaped MSNs are more readily taken up by the cells compared to the uncoated rod-shaped MSNs. Hence, this in combination with similar *P*_app_ values would mean that the copolymer coated rod-shaped MSNs with their ‘proton trap’ ability originating from PEI remain in the cell in lysosomes and multivesicular bodies to a larger extent compared to the uncoated rod-shaped MSNs. All in all, according to our interpretation, the SPR data indicates more robust uptake of the rod-shaped MSNs, especially if we compare the uncoated forms. Furthermore, the copolymer-coating clearly enhanced the uptake readily in both (spherical and rod-shaped) cases.

Overall, the results of our study show that MSNs coated with the PEG-PEI copolymers are taken up by RBE4 and MDCK II more efficiently than pure uncoated MSNs. The transport of MSN NPs across cell monolayers with high barrier properties, however, is low. In vivo, the particles were readily detected within minutes after i.v. injection in the brain vasculature, but did not cross into the brain parenchyma. This shows that our MSNs can potentially deliver their cargo across the luminal side of the blood-brain barrier, thus overcoming the first crucial challenge of brain delivery, although its subsequent distribution beyond the abluminal side will not be aided by the nanoparticles. In our in vivo study, MSN NPs did not cause any detectable damage to the BBB. This observation correlated well with the cytotoxicity studies, where no cytotoxicity was observed for any of the chosen particle designs, further stressing the applicability of the particles as a solid platform for the further design of suitable drug delivery carriers. Finally, we conclude that no MSNs we studied exhibited any substantial toxic effect, and that the robust cellular uptake of PEG-PEI copolymer-coated MSNs show that these particles may have potential in drug delivery across selectively permeable cell monolayers such as those comprising the BBB.

## Conclusions

In this study, MSNs of different morphology and surface characteristics were investigated for their potential use as drug carriers to the brain. Spherical and rod-shaped particles with the smallest dimension in the sub-100 nm range were synthesized and coated or not with PEG-PEI copolymers to facilitate permeability and uptake by cells used as a BBB model. Uptake studies indicated more efficient and robust uptake of copolymer-coated particles compared to uncoated particles. All four particle designs were safe towards the cells at the investigated concentrations, and permeability studies using an *in vitro* BBB model indicated generally low transport rates of nanoparticles across the cell layer. Microscopy evaluation confirmed the intracellular presence of copolymer-coated particles of both shapes in the cellular monolayer. Two-photon *in vivo* microscopy was applied to visualize the particles in the brain vasculature, confirming excellent *in vivo* detectability of the MSNs using multi-photon techniques. No damage to the BBB was caused by the circulating particles, further stressing the safety aspect. Thus, our study should serve as a basis for developing particles with optimized desired properties.

## Materials and Methods

### Mesoporous silica nanoparticles

Both spherical and rod shaped MSNs were synthesized according to the protocol described in reference [14], with slight modifications in order to provide higher aspect ratio for particle morphology, as well as to incorporate the fluorescent label (FITC) into the silica framework.

The chemicals used in the preparation of spherical and rod-shaped MSNs were purchased from Sigma-Aldrich. For spherical MSN synthesis, tetraethyl orthosilicate (TEOS) was employed as silica source and cetyltrimethylammonium bromide (CTAB) was utilized as pore structure directing agent (SDA) in the presence of ethylene glycol(EG),and the synthesis was performed under basic conditions. The aspect ratio of MSNs was altered by absence of ethylene glycol, slightly decreased (~5 w%) CTAB amount and elevated reaction temperature as compared to the original referred protocol. Briefly, the synthesis solution for spherical MSNs consisted of the following molar ratios: 1 CTAB/28.7 NH_3_ /3091 H_2_O/166EG/2.22TEOS, whereas for rod-shaped MSNs the molar ratio was: 1 CTAB/30.7 NH_3_ /3278H_2_O/2.37 TEOS. For the spherical MSN synthesis, the solution was kept under vigorous stirring for 2 hours at 323 K in 250 ml Erlenmeyer flask and subsequently, transferred to static conditions at 323 K overnight. For the rod-shaped MSN synthesis, the stirring and static condition temperature was elevated to333Kfor the same protocol. In order to remove the SDA, the resulting synthesis solution was centrifuged, the product was collected, extracted three times in ethanolic NH_4_NO_3_ solution and washed with ethanol. In both syntheses, in order to incorporate the fluorescent tag, fluorescein isothiocyanate (FITC)-modified aminopropyltriethoxysilanesilane APTES was mixed with the silica source before adding to the reaction solution, to provide co-condensed functionalization of FITC within the silica framework. The modification of APTES was carried out by pre-reacting FITC with APTES in 2.5 mL ethanol with a molar ratio of 2:3 and stirring for 2 h under inert atmosphere. The molar ratio between APTES and tetraethyl orthosilicate (TEOS) was kept as 1:100. The thus preserved negative surface charge was subsequently utilized for further electrostatic adsorption of PEG-PEI copolymer to the extracted MSNs.

After particle synthesis was completed, the coating was carried out for particle surface modification. The coating was proceeded by electrostatic adsorption of PEG-PEI copolymers on the particles surface in buffer solution at pH7.2 (25mM HEPES) and room temperature overnight. After the coating was completed, MSNs were centrifuged and washed carefully in order to get rid of any excess non-adsorbed copolymers in the bulk solution.

All in all the following types of spherical (S) and rod-shaped (R) MSNs were obtained, as also illustrated in Fig 1: unmodified (pure) MSNs (US-MSN and UR-MSN) and MSNs coated with a PEG-PEI copolymer (CS-MSN and CR-MSN).

The coated PEG-PEI copolymer amount on both CS-MSN and CR-MSN was calculated with the help of TGA. All the prepared MSNs were dispersed in HEPES buffer solution at pH7.2 for further investigations.

### Nanoparticle characterization

The hydrodynamic size of pristine and modified MSNs were determined by dispersing the particles in HEPES buffer solution (25 mM, pH7.2) with the concentration of 1 mg/mL and subsequent DLS measurements (Malvern ZetaSizer NanoZS, Malvern Instruments Ltd, Worcestershire, UK). The change in the ζ-potential potential values of MSNs after modification was also investigated by measurements with the same instrument in the HEPES buffer solution. A further confirmation of surface modification with PEG-PEI copolymer coating on MSNs was carried out by TGA (Netzsch STA 449F1 Jupiter, NETZSCH-Gerätebau GmbH, Germany). The TG resolution of the instrument is 0.025μg and the measurements were done under air atmosphere and in alumina crucibles, at the scanning rate of 10K/min. During the measurements, thermograms were recorded within the range of 30-850oC and the results were analyzed with the help of software Proteus 5.

The aspect ratio and the size of pristine MSNs were further investigated by scanning electron microscopy (SEM) performed with a Leo 1550 Gemini scanning electron microscope (LEO Electron Microscopy Inc., USA) operated at 3kV.The mesostructure of MSNs were investigated by transmission electron microscope (FEI Technai 12 Bio-Twin, 120 kV, FEI Company, Eindhoven, NL). TEM samples were prepared by dispersing the MSNs in ethanol, depositing them on a holey carbon coated grids and leaving them overnight drying.

### Cell culture

MDCK II (a kind gift from Prof. Yliperttula, University of Helsinki) were grown in Dulbecco’s modified Eagle’s medium (DMEM) (Sigma) supplemented as necessary to contain 10% fetal bovine serum (Thermo Scientific), 1% penicillin, 1% streptomycin (both from Sigma), 25 mM HEPES and 4 mM L-glutamine (both from Thermo Scientific). The growth conditions were as follows: 37°C, 95% relative humidity, 5% CO_2._ For transport studies, the cells (passage 21–22) were seeded at a density of 4.2x10^5^/cm^2^ onto Millicell^®^ permeable supports with 1.1 cm diameter and 1.0 μm pore size, with the pore size chosen so as to allow nanoparticle permeation; the upper and lower compartments of the permeable supports contained 0.4 ml and 1.2 ml of medium, respectively. The cells were grown for 6 days with daily medium change. For uptake and cytotoxicity studies, the cells were seeded at the same density onto 12-well and 96-well cell culture plates, respectively, and grown as described above. For SPR experiments, MDCK II cells were seeded on gold-coated coverslips and grown to confluence in the medium and under the conditions described above.

RBE4 cells (a kind gift from Prof. Ashner, Vanderbilt University) were grown on rat tail type I collagen (Millipore) in a mixture of F10 and Minimum Essential Medium (1:1) (Sigma) supplemented with 1 ng/ml bFGF, 300 μg/ml G415 and 25 um HEPES (all from Thermo Scientific). The growth conditions the same as in the case of MDCK II cells. For uptake studies, RBE4 cells were seeded in 12-well plates and 8-well Ibidi dishes for flow cytometry and confocal microscopy, respectively, and grown for 7 days with medium changed every other day. For cytotoxicity studies, RBE4 we seeded on 96-well plates and grown for 7 days as described above.

### Transport studies

On day 7 following seeding, MDCK II cells were washed twice in prewarmed phosphate-buffered saline (PBS) (Sigma) and incubated at room temperature for 25 minutes in medium without phenol red supplemented in the manner described above, with the exclusion of serum. The medium without serum and phenol red is referred below to as the transport medium. Monolayer resistance was measured with EVOM-2 (World Precision Instruments) using STX-2 electrodes sterilized by quick immersion into alcohol and equilibrated for 25 minutes at room temperature in the transport medium. Measurements were taken in triplicate, and the average of three readings was calculated. Transepithelial resistance (TER) was calculated as follows:

TER=(TERm−TERb)xA

where TER is the monolayer resistance, TERm–the average of three readings for permeable supports with cells, TERb—the average of three readings for blank permeable supports, and A–the membrane surface area (1.13 cm^2^).

Following that, permeable inserts with monolayers were transferred to new receiver plates and incubated with various MSNs at a concentration of 50 μg/ml or 20μg/ml in 0.4 ml of the transport medium. Lucifer Yellow (LY) (Sigma) was added to permeable supports in 0.4 ml of the transport medium at a concentration of 250 μM with and without 3 mM ethylene glycol tetraacetic acid (EGTA) (Sigma), in order to verify monolayer integrity and the presence of functional tight junctions, respectively (EGTA is a Ca^2+^ chelator that disrupts tight junctions). LY with or without EGTA was not co-incubated with MSNs but rather added to separate permeable supports. The number of permeable supports containing each type of MSNs, as well as LY with or without EGTA was 3 (n = 3). At time points 12, 24 and 36 hours, permeable supports were transferred to new receiver plates containing 1.2 ml of the transport medium (for samples with EGTA it additionally contained 3mM EGTA, i.e. cells were incubated in the continued presence of 3mM EGTA) and transferred back to the incubator, while the old receiver plates were stored at 4°C. At the end of the transport study, permeable supports were once again transferred to new receiver plates containing 1.2 ml of the transport medium, and TER was measured as described above. Samples were then mixed with 10M NaOH in the proportion of 1:10, i.e. to the final NaOH concentration of 1M, and left overnight on a rocker to dissolve the silica core and improve the detection limit by releasing FITC into a basic environment. Fluorescence measurements were taken using a Varioskan Flash multimode reader (Thermo Scientific) and the following excitation and emission wavelengths: 490/520 nm and 425/538 nm for MSNs and LY, respectively. Calibration solutions were prepared using the solutions initially added to permeable supports, and their fluorescence was measured simultaneously with that of the samples. Apparent permeability coefficients were calculated for each time point according to the equation suggested in [42], modified as follows:

$$Papp=\frac{V}{C_{0}\cdot A}\cdot \frac{C}{\Delta t}$$

Where V is the basolateral compartment volume, C_0_ –donor concentration, A–membrane surface area, C–concentration of the compound in the basolateral compartment, and Δt–time period. For the second and third time points C_0_ values were corrected to account for concentration changes in the apical compartment during the transport study.

### Flow cytometry

MDCKand RBE4 cells were washed twice in serum-free medium and incubated with the MSNs at concentrations of 20 μg/ml in 1 ml of serum-free mediumfor 24 hours under the conditions described above; cells not incubated with MSNs were used as controls. The number of wells containing cells incubated with each type of MSNs, as well as control cells was 3 (n = 3). After 24 hours, the cells were washed in PBS and harvested by adding 0.25% Trypsin in 1 mM ethylenediaminetetraacetic acid (EDTA) (both from Thermo Scientific). Trypsin was neutralized by serum-supplemented medium, after which the cells were centrifuged 3 times with PBS washing in-between. Extracellular nanoparticle fluorescence was quenched by incubation with Trypan Blue at a concentration of 200 μg/ml in PBS for 7 minutes. Finally, cells in PBS were taken to the flow cytometer (Beckman Coulter) for data acquisition.

### Light microscopy

For assessing MSN uptake, RBE4 cells grown on Ibidi dishes were incubated with MSNs in serum-free medium at 20 μg/ml for 24 hours, washed in PBS, fixed, incubated with Trypan blue (Sigma) at a concentration of 200 μg/ml in PBS for 7 minutes for quenching extracellular fluorescence, and mounted with SlowFade mounting medium (Thermo Scientific). Cells were visualized using a Leica SP8 confocal microscope (Leica Microsystems).

For visual evaluation of nanoparticle toxicity in transport experiments, MDCK II cells on the permeable supports from transport studies (after the final TER measurement) were washed twice in prewarmed PBS, incubated with Trypan Blue at a concentration of 200 μg/ml in PBS for 7 minutes and then washed twice in PBS. The membranes were then removed and the cells observed using a Leica DM2500 B light microscope (Leica Microsystems).

### Surface plasmon resonance measurement

Surface plasmon resonance measurements were performed with an MP-SPR 200 instrument (Bionavis, Ylöjärvi, Finland). Gold-coated SPR sensor surfaces were obtained from the MP-SPR 200 instrument manufacturer (Bionavis, Ylöjärvi, Finland). Prior to the SPR measurement, MDCK II cells were seeded on gold-coated SPR sensor surfaces and allowed to form a confluent monolayer. For the measurement, the SPR sensors with cell monolayers were inserted into the MP-SPR 200 instrument and exposed to a serum-free medium containing MSNs at 20 μg/ml. The SPR response was monitored for 175–300 minutes at room temperature.

### *In vivo* imaging experiments

All experiments on animals were performed with accordance to local guidance for animal care (The Finnish Act on Animal Experimentation (62/2006)). Animal license (ESAVI/2857/04.10.03/2012) from local authority (ELÄINKOELAUTAKUNTA-ELLA) was obtained and included all procedures used in in vivo imaging part of this study.

One female two-month old mouse was used for in vivo experiment. The mouse was ordered from Harlan—commercial supplier of laboratory animals. It was kept in individual cage in the certified animal facility and provided with food and water ad libitum. 12h light/12h dark cycle was applied.

For imaging of nanoparticles in the brain vasculature, a 5 mm cranial window was implanted in a C57BL/6 mouse. During preparation to surgery a subcutaneous injection of 0.1% lidocaine was used to reduce local pain at the incision site. Mouse was anaesthetized (i.p.) with mixture of ketamine (80 mg/kg) and xylazine (10 mg/kg). Ketamine/xylazine mixture was re-administered every hour if necessary at half-the-original dose. Body temperature was maintained using a heating pad. For cranial window formation, a ~3×3 mm round craniotomy was performed over the somatosensory cortex. Cranial bone was carefully removed, while dura mater remained intact. The brain was then covered with sterile cortical buffer (125 mM NaCl, 5 mM KCl, 10 mM glucose, 10 mM HEPES, 2 mM CaCl2 and 2 mM MgSO4 in distilled H2O), and a 5 mm diameter No. 1.5 glass coverslip (Electron Microscopy Sciences, USA) was placed over the window and sealed using dental cement.

Animal was imaged immediately after window preparation with the FV1000MPE two-photon microscope (Olympus, Japan) using the 25X water immersion high NA objective specially designed for in vivo two-photon imaging. Stacks of images were collected with vertical step of 3 μm (total depth of stack 120 μm). Imaging fields covered 500 × 500 μm2 of cortical space in XY coordinates. MSNs were injected i.v. into the tail vein of a mouse. Imaging was repeated 48 hours after the first imaging session. After the imaging, the mouse was sacrificed by CO2 inhalation.

### Cytotoxicity studies

Cells were washed twice in serum-free medium and incubated with various MSNs at concentrations of 100, 50, 20 and 10 μg/ml in serum-free medium for 36 hours under the conditions described above. Untreated cells were used as the negative control. Cells treated with 0.1% Triton X were used as the positive control. The number of wells containing cells incubated with each type of MSNs was 3 (n = 3). After 36 hours, a cell viability reagent (Alamar Blue from Thermo Scientific) was added to the wells. After an incubation period recommended by the manufacturer for each reagent, measurements were read using a plate reader (Tecan Infinite M200 from Tecan Group Ltd).

### Data processing and analysis

Statistical analysis was performed with Student’s test using SPSS v17. SPSS v17 was also used to plot data from transport, flow cytometry and cytotoxicity studies, as well as various model validation experiments. Data from flow cytometry studies were plotted on histograms using Kaluza software (Beckman Coulter). Confocal microscopy images were read and prepared for publication using ImageJ image processing software. Data were expressed as M±2xSEM, where M is the mean and SEM is the standard error of the mean. Statistical analysis was only applied to novel results, i.e. those concerning MSN permeability, uptake and cytotoxicity.

## Supporting Information

S1 Fig Lower end of the calibration curves of PEG-PEI-coated spherical MSNs in serum-free medium. Instrument readings denote fluorescence intensity of NP-PEG-PEI solutions made by serial dilution from stock solutions after their ultrasonication for 30 minutes with shaking in-between. A. Untreated PEG-PEI-coated spherical MSNs in serum-free medium. B.PEG-PEI-coated spherical MSNs in 1M NaOH, after overnight rocking on a bench rocker.
(TIF)

S2 Fig TER measurement in permeable support wells containing MDCK II cells incubated for 36 hours with various types of MSNs at a concentration of 50 μg/ml, as well as 250 μM LY with or without 3 mM EGTA in serum-free medium. Measurements were taken before and after transport studies. The sample size n = 3, and TER measurements were taken in triplicate with subsequent averaging. Data shown as M±2xSEM.
(TIF)

S3 Fig Transport of LY across MDCK II monolayers on permeable supports. LY applied at a concentration of 250 μM in serum-free medium. MDCK II monolayers were incubated with LY in or without the constant presence of 3 mM EGTA. The sample size n = 3. Data represent LY Papp at 12, corrected for the loss of LY in the upper compartment of permeable supports, and is shown as M±2xSEM.
(TIF)

S4 Fig SPR signal response showing the addition of DMSO. (TIF)

S1 File Supporting information file. This file contains additional information on the experimental procedures, mostly related to model validation and improvement.
(DOCX)

S1 Video Uptake of coated spherical MSNs by MDCK II cells. Live-cell imaging. The cells were pre-incubated with Cellmask Deep Red Plasma Membrane Stain for 5 minutes in cell culture medium, and then incubated with coated spherical MSNs at 20 μg/ml in live cell imaging medium for 1 hour while being imaged.
(AVI)
