# Supplementary figures and images for: Feasibility Study of the Permeability and Uptake of Mesoporous Silica Nanoparticles across the Blood-Brain Barrier
Source: PLoS One. 2016 Aug 22;11(8):e0160705. doi: 10.1371/journal.pone.0160705 (PMC4993362; doi:10.1371/journal.pone.0160705)

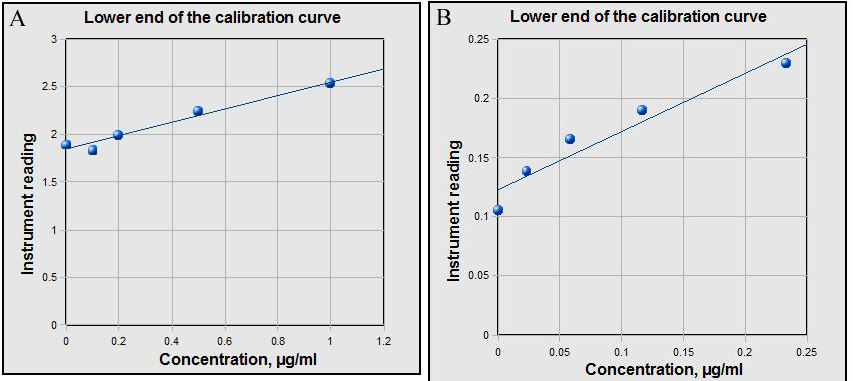

Supplement: S1 Fig — Instrument readings denote fluorescence intensity of NP-PEG-PEI solutions made by serial dilution from stock solutions after their ultrasonication for 30 minutes with shaking in-between. A. Untreated PEG-PEI-coated spherical MSNs in serum-free medium. B.PEG-PEI-coated spherical MSNs in 1M NaOH, after overnight rocking on a bench rocker. (TIF) [file pone.0160705.s001.tif]

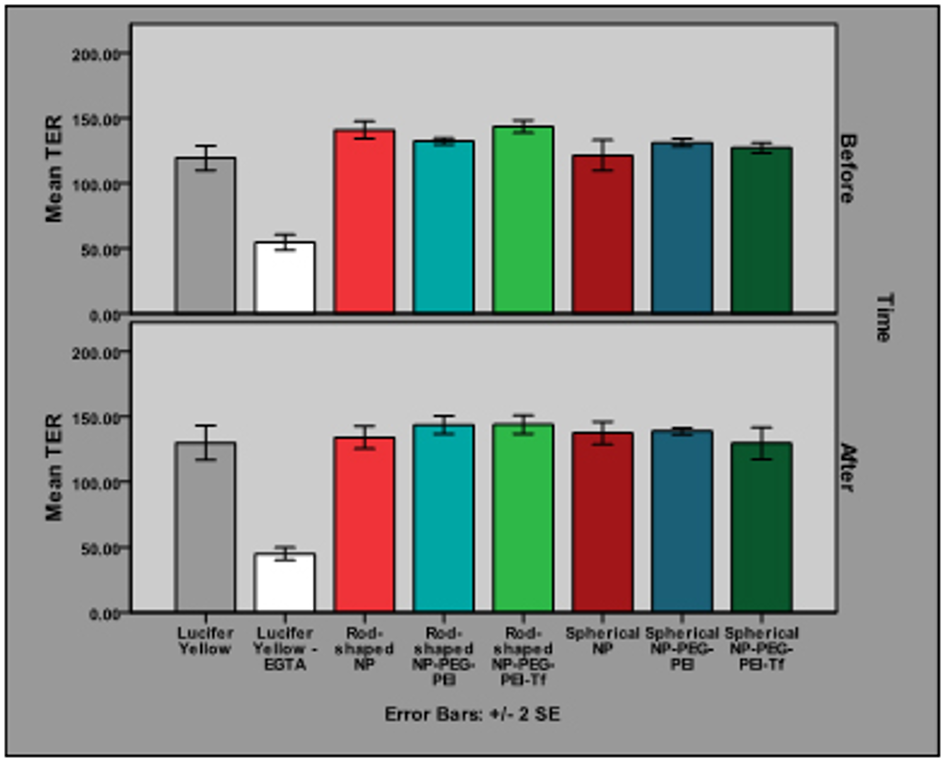

Supplement: S2 Fig — Measurements were taken before and after transport studies. The sample size n = 3, and TER measurements were taken in triplicate with subsequent averaging. Data shown as M±2xSEM. (TIF) [file pone.0160705.s002.tif]

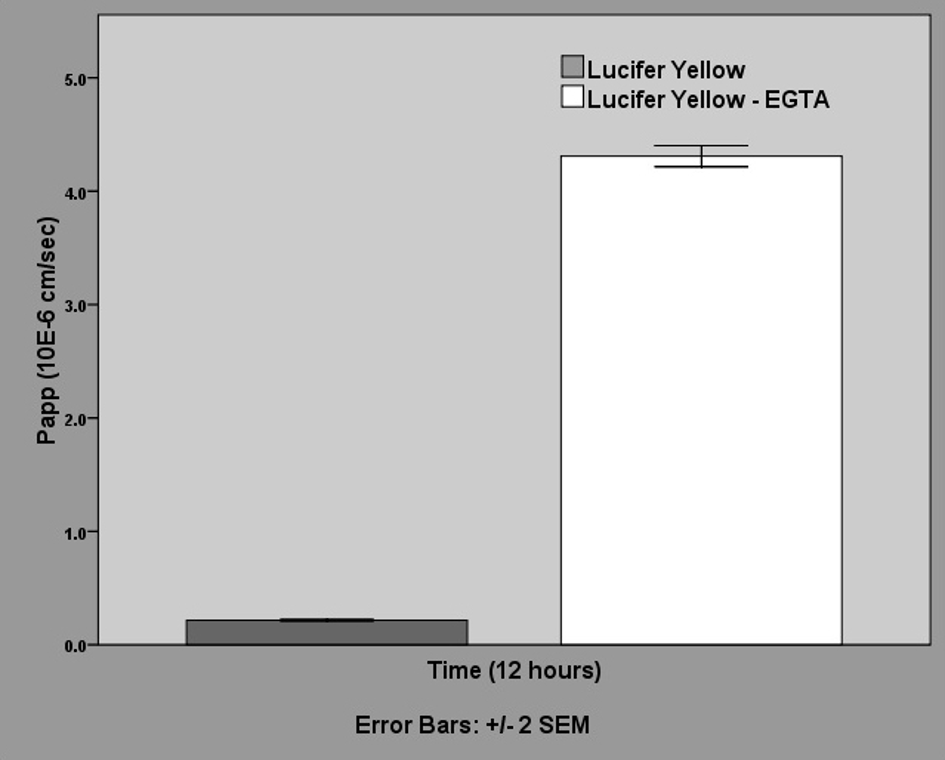

Supplement: S3 Fig — MDCK II monolayers were incubated with LY in or without the constant presence of 3 mM EGTA. The sample size n = 3. Data represent LY Papp at 12, corrected for the loss of LY in the upper compartment of permeable supports, and is shown as M±2xSEM. (TIF) [file pone.0160705.s003.tif]

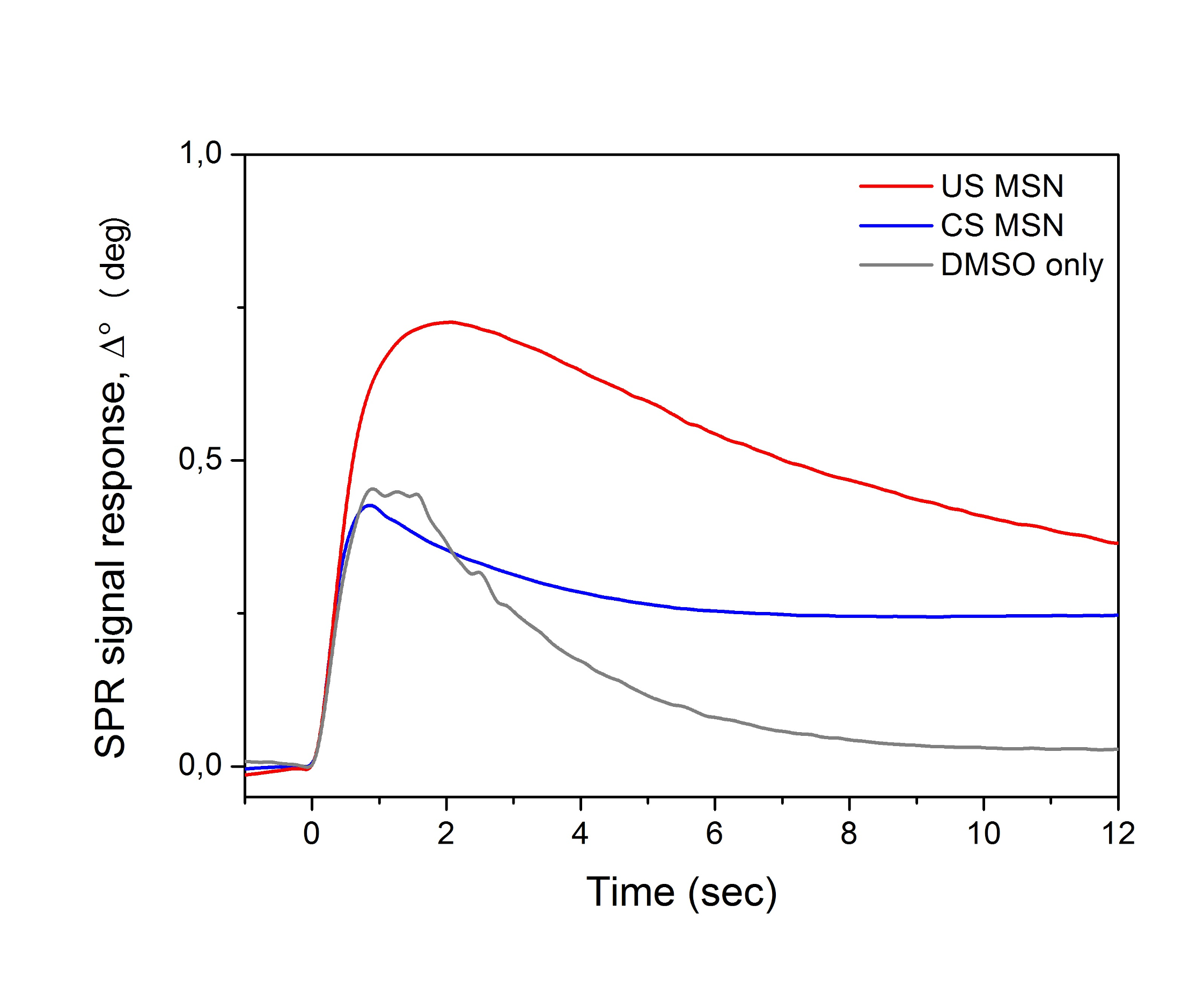

Supplement: S4 Fig — (TIF) [file pone.0160705.s004.tif]
