# Supplementary material for: Feasibility Study of the Permeability and Uptake of Mesoporous Silica Nanoparticles across the Blood-Brain Barrier
Source: PLoS One. 2016 Aug 22;11(8):e0160705. doi: 10.1371/journal.pone.0160705 (PMC4993362; doi:10.1371/journal.pone.0160705)
Supplement: S1 File — This file contains additional information on the experimental procedures, mostly related to model validation and improvement. (DOCX) [file pone.0160705.s005.docx]

**Supporting information**

Model validation and improvement

In order to validate the in vitro BBB model, we assessed the model’s monolayer integrity by measuring TER and LY transport across MDCK II monolayers grown on permeable supports. The presence of functional TJs was assessed by measuring said integrity markers in the presence of EGTA that opens TJs, causing a drop in TER and increased paracellular permeability of LY. To improve the detection limit of MSNs and, therefore, be able to conduct transport studies at lower and safer MSN concentrations, we treated MSN samples with 1M NaOH that dissolves MSNs and releases the attached FITC into a basic environment, enhancing FITC fluorescence.

As shown in S1 Fig. A and S1 Fig. B, the detection limit of PEG-PEI-coated spherical MSNs without NaOH treatment is approximately 0.2 µg/ml. The addition of NaOH, however, dissolves the silica core and releases FITC into a basic environment that enhances its fluorescence, thus improving the detection limit to approximately 0.02 µg/ml.

As shown in S2 Fig., TER values in the wells containing MDCK II monolayers incubated for 36 hours with MSNs at a concentration of 50 µg/ml are approximately the same before and after transport studies and are not considerably different 1) from the TER values of monolayers incubated with 250 µM LY, and 2) from each other, which indicates lack of monolayer disruption to the extent that would be detectable by TER measurement. The addition of 3 mM EGTA sharply reduced the TER values, indicating the presence of functional tight junctions.

As shown in S3 Fig., the permeability of LY across MDCK II monolayers remains very low (approximately 0.2x10-6 cm/sec) throughout the study, indicating monolayer integrity. In the presence of 3 mM EGTA, the permeability of LY is significantly increased, further confirming the presence of functional TJs.
